# Anaesthetic Management of A Child with Multiple Congenital Anomalies Scheduled for Cataract Extraction

**Published:** 2009-12

**Authors:** Kalpana Kulkarni, Sunetra Deshpande, Ismail Namazi

**Affiliations:** 1 Asso. Prof, Department of Anesthesiology, Dr.D.Y.Patil Medical College , Kadamwadi, Kolhapur, Maharashtra; 2 Lecturer, Department of Anesthesiology, Dr.D.Y.Patil Medical College , Kadamwadi, Kolhapur, Maharashtra; 3 Prof, Department of Anesthesiology, Dr.D.Y.Patil Medical College , Kadamwadi, Kolhapur, Maharashtra

**Keywords:** Congenital subglottic stenosis, Airway malacia, Anesthesia, Difficult intubation

## Abstract

**Summary:**

In infants & children variety of conditions and syndromes are associated with difficult Airway. Anaesthetic management becomes a challenge if it remains unrecognized until induction and sometimes results in disaster, leading to oropharyngeal trauma, laryngeal oedema, cardiovascular & neurological complications. A 4-month-old child with multiple congenital anomalies was posted for cataract extraction for early and better development of vision. He had history of post birth respiratory distress, difficulty in feeding, breath holding with delayed mile stones. He was treated as for Juvenile asthma. This child was induced with inhalation anaesthesia. There was difficulty in laryngoscopic intubation and could pass much smaller size of the tube than predicted. He developed post operative stridor and desaturation. The problems which we faced during the anaesthetic management and during postoperative period are discussed with this case.

## Introduction

Airway abnormality in a child may remain undetected in the presence of other multiple congenital defects. Wells et al reported association of subglottic stenosis, shortened trachea, fewer tracheal rings, shorter glottis carinal length in significant percent of the patients with several congenital malformation syndromes. Down's syndrome has the higher incidence of such associated airway abnormalities^1^. Subglottic stenosis is the narrowing of the lumen at the level of cricoid <4mm in full term infant or <3.5mm in a premature. It may be congenital or acquired^2^. It is graded (I–IV) for mild to severe stenosis according to the tube size accommodated^3^. Boogaard-2005 reported incidence of primary airway malacia to be at least 1 in 2100 infants. The presenting clinical features of children with airway malacia are variable, with overlapping features of allergic asthma^4^.The problem is structural abnormality and immaturity of tracheal rings allowing collapse of the trachea. Diffuse malacia of airway of congenital origin improves by age of 6-12 months. Based on histological, endoscopic and clinical presentation tracheomalacia is classified into three types^5^. Tracheomalacia -Intrinsic Type -I is primarily congenital, known to be associated with cardiovascular anomalies, developmental delay, tracheo-oesophageal fistula, laryngomalacia. The prevalence of gastro-oesophageal reflux is also very high in these infants^6^. Type -II tracheomalacia is secondary to the compression by extrinsic anomalies and Type -III is acquired following long term intubation or tracheostomy^5^. Airway malacia are difficult to diagnose and are known to be responsible for considerable morbidity, mortality and significant difficulties in the operation theatre and intensive care unit^7^. Anaesthesia becomes a challenge in a child with congenital anomalies involving airway, this may lead to disastrous situation during the course of anaesthesia. We are presenting a child having multiple anomalies with undiagnosed airway defect that was posted for congenital cataract extraction, with the aim to discuss the problems which we experienced during induction/ intubation, maintenances as well as during immediate and late post operative period.

## Case Report

A 4-month-old male child weighing 4.5kg, height of 55cm, having congenital bilateral cataract was posted for cataract extraction of right eye. It was IInd full term normally delivered baby, cried well after birth but later developed respiratory distress which required neonatal intensive care for 15 days. Since birth he had excessive salivation, noisy breathing on and off, with breath holding during crying and feeding without cyanosis. He had repeated attacks of hacking cough and distress which required intensive treatment. Once surgery was postponed for the same reason. Diagnosis of recurrent lower respiratory tract infection with juvenile asthmatic exacerbations were made. Nothing specific in his family history and the milestones were moderately delayed.On investigation his haemoglobin was 9.8gm% with normal coagulation profile. X-ray chest depicted cardiomegaly with normal airway and lung fields.2D- echocardiography revealed mild valvular pulmonary stenosis. The electro- cardiogram was normal. Ultrasonography of brain revealed 4x3mm cyst in choroid plexus of right frontal horn. He was negative for rubella virus antibodies. On general examination he was afebrile, acyanotic, anicteric & dysphonic with weak cry. The respiratory rate (RR) of 48 /min with minimal sub costal, sternal in drawing. The neck mobility, head and tongue sizes were normal to his age. On oral examination soft palate and base of the uvula was seen. The heart rate (HR) was 140/min, with short systolic murmur and the chest was clear. There was no spinal deformity, muscle tone and the reflexes were normal. Pupils were normal in size, reaction and the visions were absent.

Informed consent was obtained from the parents. Prophylactic antibiotic, nebulisation with salbutamol, hydrocortisone 4mg.kg^−1^ intravenously (IV) was given a day before and on the day of operation. Child was kept nil by mouth for 4 hours prior to the surgery. On pulse oximeter his SpO2 on air was 98%. Anaesthetic drugs & equipments were checked and premedicated with glycopyrrolate 0.01mg.kg^−1^, midazolam 0.05 mg.kg^−1^ IV. Patient was induced with halothane up to 2.5% in 50% of N2O and O2 with assisted ventilation and adequate depth was achieved (jaw relaxed, flaccid arms / hands with centrally fixed pupils).The laryngoscopy was performed with curved / straight blade No.1. Larynx could not be visualized. Intubation was attempted twice with size 3.5/3 mm (Internal diameter) plain endotracheal (ET) tube but failed to intubate. Mask ventilation was ensured and laryngoscopy performed under the effect of succinylcholine 1mg.kg^−1^.With external laryngeal pressure, posterior commissure was partly visible. Intubation succeeded with No.2 plain ET tube with resistance. The tracheal placement was confirmed and maintained on 50% O2 in N2O and halothane by assisted ventilation. Surgery lasted for 30min. Intra operatively vitals were stable. The child was extubated after full recovery of consciousness. SpO2 on air was 98%. After a period of 20 min in the recovery he developed mild stridor, SpO2=90% with RR of 52/ min.

**Fig 1:**
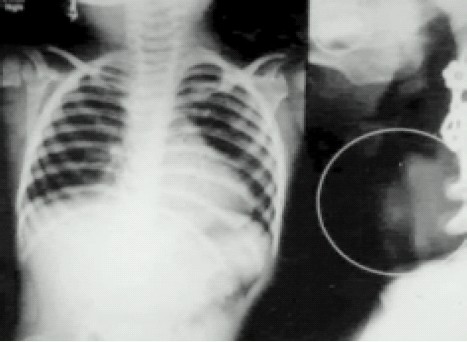
X-ray chest neck

**Fig 2:**
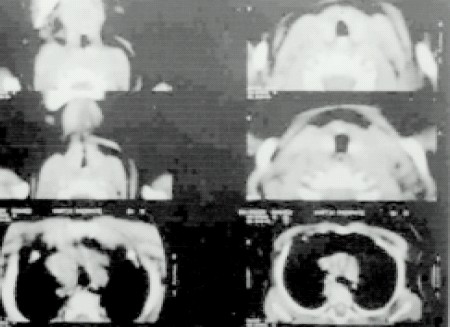
CT scan upper airway

**Fig 3:**
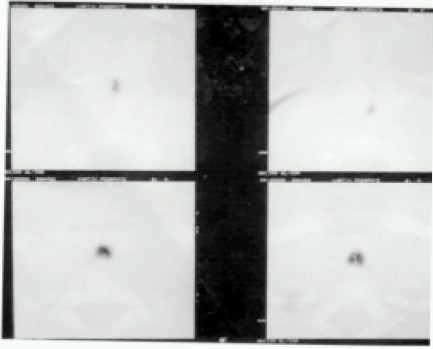
CT scan upper airway

Considering intubation attempts and snuggly fitted tube we avoided re intubation and managed the situation with lateral positioning, jaw thrust, O2, IV dexamethasone 2mg, hydrocortisone 25mg.O2 saturation improved to 96% with clear chest and normal vital records. Within an hour in pediatric intensive care unit (PICU), he re-developed biphasic stridor, ronchi and labored respiration. SpO2 dropped to 88%, RR was 66/min and HR-170/min.He was conscious, crying and struggling to move. He was nebulised with salbutamol, IV steroids, O2 by mask. He responded to the treatment slowly over 5days.The parents were informed about the difficult intra and post operative course, also the need of further investigations to rule out any associated airway problem. Considering the urgency of second operation for better vision, the risk of anaesthesia and if needed tracheostomy with intensive care was explained. The child was reposted for surgery of left eye, after 15 days. But due to poor economical status and refusal for the risk consent, surgery was postponed. The child reappeared after 8months for the reason of poor vision and status quo respiratory complaints. X-ray chest PA/Lat.view of neck (Fig. 1) and CTscan of the upper airway (Figs. 2 and 3) and chest was done. Post cricoid narrowing was detected. Pediatrician reviewed and concluded subglottic stenosis with? Tracheomalacia.The child was 1year old, weight 8kg, hight-75cm and acyanotic.His RR was 40/min with minimal sub costal in drawing. HR- 124/min and soft systolic murmur +. Mental retardation +, Mile stones = 10months. Airway status- Mallampati class III. Informed high risk consent for anesthesia and tracheostomy were obtained.

Pre-operatively IV steroids, nebulization with bronchodilator was given. Premedicated with. intramuscular glycopyrrolate 0.01mg.kg^−1^ and midazolam 0.05 mg/kg. Besides routine, tracheostomy tray and emergency transtracheal ventilation set were kept ready. The pediatric size LMA or fibreoptic scope were not available with us. We planned for induction & intubation under O2, N2O, and halothane anaesthesia. But we failed to achieve the desired level of induction after 20–30 min.As mask ventilation was possible, IV propofol 1mg.kg^−1^ and succinylcholine 1.25 mg.kg^−1^ was injected. On laryngoscopy with external laryngral pressure the highly placed larynx was seen as a small hole, with ill defined margins. 2% lidocaine sprayed over the larynx. On third attempt intubation was possible with No.2.5 ET tube on stylet. The tube position confirmed and the chest was clear. A dose of atracurium 0.5mg.kg^−1^ was given. Peribulbar block was achieved with 1.5ml of 1% lidocaine. After some time there was gradual fall in SpO2 up to 88% over 10 min, so halothane was cut off and supplemented with IV propofol 0.5 mg/kg. SpO2 improved up to 95%, end tidal CO2 of 35–40 mmHg. Moderate resistance to IPPV was present throughout the surgery. Reversal was achieved with neostigmine 0.05 mg/kg & glycopyrrolate 0.02 mg.kg^−1^. The child was extubated after full recovery and SpO2 on air was 98%.Immediately after extubation he again developed stridor, dyspnoea with desaturation. He was managed with O2, jaw thrust and nebulisation with steroids and bronchodilators and was observed inside the operation theatre for one hour and then shifted to PICU. Later in PICU he had similar respiratory distress within two hours and required intensive treatment with inhalational / parenteral steroids and bronchodilators for 6 postoperative days.He was shifted to the wards on 7^th^ post operative day and discharged on 10^th^ day.

## Discussion

Multiple congenital defects when associated with airway abnormalities may present with mixed picture of respiratory symptoms. Airway malacia associated with bronchopulmonary dysplasia may be the reason for long term intensive care with tracheostomy and ventilatory support^7^. Laryngomalacia is the commonest cause of stridor in young infants (60%) after the post extubation stridor^8^. In most of the cases it disappears after 18–24 months. Congenital subglotic stenosis presents with biphasic stridor and picture of recurrent croup with dyspnoea. In severe stenosis there may be irritability, tachypnoea, cyanosis. Tracheomalacia has been implicated for one of the cause of sudden infant death syndromes^9^. In our case during first instance, the history and routine examination was inconclusive of air- way anomaly and child was diagnosed and treated as for juvenile asthma. The anaesthetic experience of difficult laryngoscopy, intubation with smaller size of tube and stormy postoperative course raised the suspicion of associated airway anomaly. During second anaesthethesia the X-ray / CT diagnosis of subglottic stenosis was kept in mind. But with non availability of safe alternative technique like fibreoptic intubation, we planned for the inhalation anaesthesia with intubation and if required tracheostomy. Inability to induce with inhalation agent which was mostly due to smaller size of the laryngeal inlet (? laryngomalacia or web). Besides, intubation with much smaller size snuggly fitting tube (2.5, predicted size 4) and post extubation stridor were the confirmatory signs of associated subglottic stenosis. The incidence of post intubation croup is 1%, which is commonly due to tight fitting ET tube^10^. Laryngeal spray with racemic epinephrine is helpful as a rescue measure in severe stridor but the risk of rebound oedema still remains^11^. Delayed extubation or elective tracheostomy was avoided for the reason of associated complications like post extubation subglottic oedema and the risk of post tracheostomy stenosis. There are reports of using LMA safely to avoid intubation and subsequent complications like coughing, post extubation croup and airway collapse^12^. With LMA there is a risk of airway obstruction during general anaesthesia with spontaneous respiration in patients with collapsible airway, so the controlled ventilation is advisable^13^. Recently reports of using Cobra PLUSTM, an extra glottic airway device found to be safe in a child with tracheomalacia^14^. Our patient with stridor, dysphonia & recurrent respiratory distress with prolonged post operative course, needs further investigations like flexible laryngo-bronchoscopy, dynamic fluoroscopy/ CT/ MRI and pulmonary function test to confirm the association of congenital airway malacia.

The paediatric airway itself and when associated with airway malacia/stenosis in a child with multiple congenital syndromes, have higher incidence of difficult intubation as well as post extubation complications. The associated airway anomaly may remain unrecognized or undiagnosed preoperatively. So with the history of recurrent respiratory tract infections requiring intensive care, the possibility of the narrowing of air passage and post extubation complications like stridor or respirstory distress must be kept in mind. The intra operative difficult course may be followed by dreadful complications. Hence one should be prepared with the plan for failed intubation /ventilation and for the post operative consequences.
